# Microbial metabolic activity in Amazon floodplain forest and agricultural soils

**DOI:** 10.3389/fmicb.2023.1144062

**Published:** 2023-05-24

**Authors:** Dayane J. Barros, Glauber A. Carvalho, Miriam G. de Chaves, Luiz S. Vanzela, Dora Inés Kozusny-Andreani, Emerson A. Guarda, Vania Neu, Paula B. de Morais, Siu M. Tsai, Acacio A. Navarrete

**Affiliations:** ^1^Graduate Program in Biodiversity and Biotechnology (BIONORTE), Federal University of Tocantins (UFT), Palmas, Brazil; ^2^Faculty of Engineering, Architecture and Urbanism, Federal University of Mato Grosso do Sul, Campo Grande, Brazil; ^3^Cell and Molecular Biology Laboratory, Center for Nuclear Energy in Agriculture, University of São Paulo (USP), Piracicaba, Brazil; ^4^Graduate Program in Environmental Sciences, University Brazil, Fernandópolis, Brazil; ^5^Federal Rural University of Amazonia (UFRA), Belém, Brazil

**Keywords:** substrate type, environmental factor, Biolog ECO plates^®^, drought and flooding, functional diversity

## Abstract

Microorganisms play an essential role in ecosystem functions. An increasingly used method for conducting functional analyses of a soil microbial community is based on the physiological profile at the community level. This method allows the metabolic capacity of microorganisms to be assessed based on patterns of carbon consumption and derived indices. In the present study, the functional diversity of microbial communities was assessed in soils from seasonally flooded-forest (FOR) and -traditional farming systems (TFS) in Amazonian floodplains flooded with black, clear, and white water. The soils of the Amazon floodplains showed differences in the metabolic activity of their microbial communities, with a general trend in activity level of clear water floodplain > black water floodplain > white water floodplain. The redundancy analysis (RDA) indicated that soil moisture (flood pulse) was the most important environmental parameter in determining the metabolic activity of the soil microbial communities in the black, clear, and white floodplains. In addition, the variance partitioning analysis (VPA) indicated that the microbial metabolic activity of the soil was more influenced by water type (41.72%) than by seasonality (19.55%) and land use type (15.28%). The soil microbiota of the white water floodplain was different from that of the clear water and black water floodplains in terms of metabolic richness, as the white water floodplain was mainly influenced by the low substrate use during the non-flooded period. Taken together, the results show the importance of considering soils under the influence of flood pulses, water types, and land use as environmental factors when recognizing functional diversity and ecosystem functioning in Amazonian floodplains.

## 1. Introduction

Soil is a dynamic and complex ecosystem whose functionality is related to the established link between environmental factors and resident microbial communities. Microorganisms play an essential role in ecosystem functions and production (Shah and Belozerova, 2009). Monitoring a microbial community and understanding the mechanisms that regulate the microbial diversity of the soil, as well as its functional characteristics, may constitute a methodology for understanding basic and applied ecological contexts (Frac et al., 2012).

Microbial ecologists are increasingly recognizing functional diversity as a link between biodiversity patterns and ecosystem functioning, determining trophic relationships and interactions between micro-organisms, their role in biogeochemical cycles, and feedback to environmental change (Escalas et al., 2019). However, information on metabolic functions at different scales is still limited. One way to quantify these functions is the use of BIOLOG™ EcoPlate method (Biolog Inc., Hayward CA, United States), which provides information on the overall and metabolic profiles of soil microbial communities (Wang et al., 2021).

Physiological profiles at the microbial community level revealed with BIOLOG™ EcoPlate can fundamentally evaluate the ability of soil microbial communities to metabolize a range of organic substrates with different structural complexities (Hueso et al., 2012). This technique was designed especially for microbial ecology studies, provides a metabolic response pattern of the microbial population in mixed cultures or environmental samples (Gryta et al., 2014), and is based on incubation in a microplate containing different carbon sources with replicates.

Undoubtedly, the most striking variations in forests in the Amazon rainforest are seasonal floods (Flores et al., 2017) and subsequent processes. The large Amazon floodplains are formed by a mosaic of habitats governed by the flood pulses of rivers. In these environments, there is a wide diversity of habitats due to several linked factors, such as the duration, amplitude, frequency, and predictability of floods (Junk et al., 2015). A better understanding of the relationships established in wetlands is necessary in light of the impacts of changing land use and occupation on ecosystem services provided by wetlands. Understanding the mechanisms that control the responses of the microbial community within the context of the natural variation to which the environments are exposed is essential for understanding environments that have high ecological uniqueness.

Amazonian waters are classified into three distinct biogeochemical categories based on attributes such as dissolved nutrients, sediment type, transparency, and acidity (Bogotá-Gregory et al., 2020) and are named based on their color (black, clear, and white). Black waters have acidic pH values, high concentrations of humic substances, and low concentrations of suspended sediment. In turn, clear waters have low suspended sediment concentrations and high transparency levels, and white waters, also known as muddy waters, are considered neutral, with low transparency levels and high loads of fertile alluvial suspended sediment (Junk et al., 2011; Bogotá-Gregory et al., 2020).

At the terrestrial–aquatic interface, the water regime has significant control over the transformation of microbial carbon (C) (Weise et al., 2016). Therefore, microorganisms can react specifically to the entry of organic substances. If the degree of oxygenation of a system change, then flooding of the soil can change the intensity and mode of carbon use.

Because of this lack of information about the microbial metabolic community potential in Amazonian floodplains under different water types, and the substantial effects that seasonality (flood pulse) and land use may have on the metabolic capabilities of microbes within this transition systems between aquatic and terrestrial ecosystems, we determined the physiological profiles at the soil microbial community level using BIOLOG™ EcoPlate method to provide information on the metabolic functional diversity of soil communities under flood and non-flood conditions in three floodplains with different water types in the Amazon, black (Negro River), clear (Tocantins River), and white (Solimões River), and land uses (forest and traditional farming systems). Two hypotheses were evaluated: (i) the ability of soil microbial communities to metabolize organic substrates is affected by the flood pulses in Amazonian floodplains and (ii) the community-level physiological profiling based on carbon substrate utilization vary according to the types of water in the Amazon floodplains.

## 2. Materials and methods

### 2.1. Sampling sites

**Figure 1:**
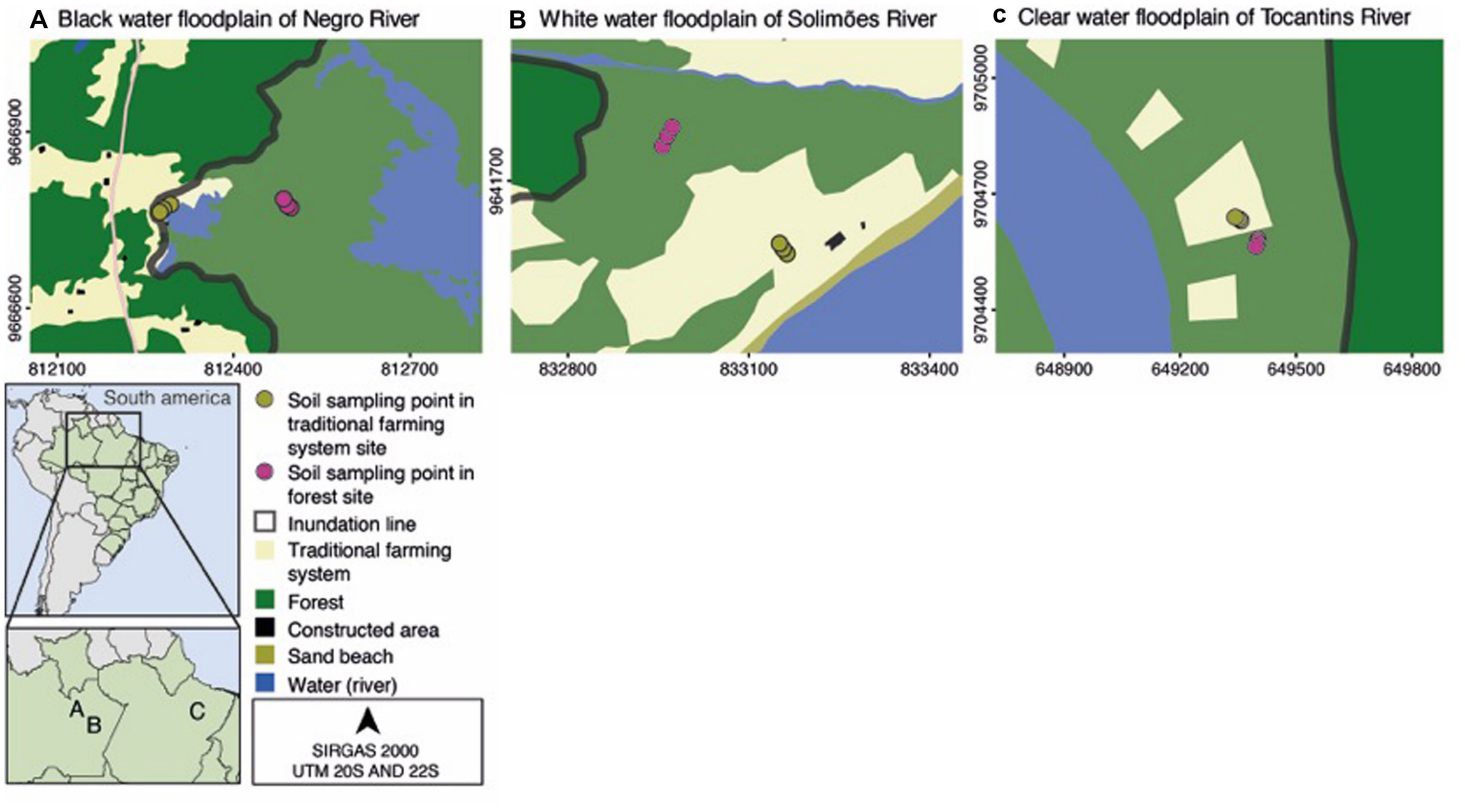
Sampling sites in the Brazilian Amazon, land use and flood line in the three study areas seasonally flooded with “black water **(A)**, white water **(B)**, and clear water **(C)**. Adapted from “Active methane processing microbes and the disproportionate role of NC10 phylum in methane mitigation in Amazonian floodplains”, by Bento et al., (2021).

Six sampling sites were identified as FOR and TFS and characterized using satellite images from Shuttle Radar Topography Mission (SRTM) at a resolution of 1 arc-second (30 m), Landsat 8 satellite, Google Earth with Universal Transverse Mercator (UTM) projection and Datum SIRGAS (datum 22S e 20S), visit in the field and geoprocessing tools in QGIS v.2.18 ‘Las Palmas’. These sites were located into three areas of floodplain periodically flooded with black, white and clear water from large Amazonian rivers and used for soil sampling in the contrasting high and low water seasons (at the drying—October 2017 and flooding—May 2018 regimes of the floodplains) (Figure 1).

Undeformed soil cores were collected at each of the six sampling sites in contrasting periods of the hydrograph (drought and flood). The samples consisted of six combinations of “water type” vs. “land-use type” in non-flooded (October 2017) and flooded (May 2018) periods. The sampling sites at each site are affected by a long-term flood pulse (more than 5 months). The study sites were located in the black water floodplain of the Negro River (3°00′41”S and 60°11′15”W), the clear water floodplain of the Tocantins River (2°40′51”S and 49° 39′05”W) and the white water floodplain of the Solimões River (3°13′50”S and 59°59′26”W). The predominant climate in this region is humid tropical, classified as ‘Afi’ (Koppen classification) with average annual temperatures above 26°C, annual rainfall of approximately 2000 mm, intense light incidence, high air humidity, and low wind speeds (Alvares et al., 2013).

The primary forest, i.e., a forest without significant disturbance, was adjacent to a traditional farm system in all three floodplains. In these traditional farm systems, subsistence agriculture based on watermelon, okra, and rambutan has been practiced in the floodplain of the black waters of the Negro River and the cultivation of tomato and cassava has occurred in the floodplain of the white waters of the Solimões River (Bento et al., 2021). In the clear water river floodplain, the traditional farm system is a cocoa-based agroforestry system.

### 2.2. Soil sampling

Soil samples were collected from the forest sites (FOR) and traditional farm systems (TFS) located in each of the three Amazon floodplains (Figure 1). At each site, soil samples were collected at three sampling points equidistant (14 m) from each other. The soil samples were collected with the aid of an aseptic cylindrical tube (soil layer of 0–15 cm in diameter and 10 cm in diameter) during the non-flooded period (NF). Under the flood regime (F) of the floodplains, cores of underwater soil were collected (soil layer of 0–15 cm and 10 cm in diameter) using a universal manual sediment sampler, with an adjustable core of 0–15 cm (Aquatic Research Instruments, ID, United States). Soil cores collected in both drying and flood regimes of the floodplain were 500 g capacity plastic bags properly identified with the sites and seasonal period.

### 2.3. Analysis of the physiological profile at the community level

Community-level physiological profiles were determined using a BIOLOG™ EcoPlate (Biolog Inc., Hayward, CA, United States). The BIOLOG™ EcoPlate consisted of 96 wells containing 31 carbon sources (6 amino acids, 2 amines, 10 carbohydrates, 7 carboxylic acids, 2 phenolic compounds, and 4 polymers) and one blank (control), in triplicate. The soil samples were subjected to preparation before inoculation in the plate wells. For this purpose, 1 g of fresh soil was added to 9 mL of sterile 0.85% NaCl (10^−1^ dilution), and the mixture was stirred at 150 rpm at a temperature of 25°C for 30 min (Mendes et al., 2019). Serial dilutions were made of each sample, and a 150 μL aliquot (10^−3^ g mL^−1^ dilution) was added to the BIOLOG ™ EcoPlate. The plates were incubated at 25°C, and the absorbance was read at 540 nm (Vinther et al., 2001, 2003; Feckler et al., 2016) every 24 h for 168 h in a microplate reader (LMR-96, Locus, SP, Brazil). Soil collected from each of the three sampling points at each sampling site in the three different floodplains was inoculated into a single BIOLOG™ EcoPlate. The absorbance at 540 nm (OD_540_) was measured immediately after inoculation of the plates (*t* = 0). The optical density (OD) values were subjected to data corrections, including first subtracting the value from the control well (water only) followed by subtracting the initial OD value from each well measured immediately after filling the wells with the soil suspension (*t* = 0) to eliminate the effect of soil particles on the reading of the OD values. The negative values were defined as zero (Mendes et al., 2019). The microbial activity in each microplate, expressed as the average metabolic activity (*Average Well Colour Development*—AWCD), was determined as follows:


$$AWCD=\sum \left(C_{i}-R\right)/31$$

where *R* is the absorbance of the control well (containing water instead of the *C* source) and *C_i_* is the absorbance of the plate inoculated with a *C* source. For AWCD, a value of 0 was assigned when *C_i_*−*R* < 0. The BIOLOG™ EcoPlate readings at 168 h (Koner et al., 2021; Song et al., 2021; Wang et al., 2021; Deng et al., 2022) were used to calculate metabolic richness. This measure corresponds to the number of oxidized *C* substrates.

To simplify the direct comparison, six guilds of substrates were grouped according to (Sala et al., 2008) amino acids (L-arginine, L-asparagine, L-phenylalanine, L-serine, glycyl-L-glutamic acid, and L-threonine), amines (phenylethylamine and putrescine), carboxylic acids (D-galacturonic acid, D-malic acid, itaconic acid, pyruvic acid methyl ester, D-glucosaminic acid, alpha-ketobutyric acid, and gamma-aminobutyric acid), carbohydrates (D-mannitol, glucose-1-phosphate, D,L-alpha-glycerol phosphate, beta-methyl-d-glucoside, D-galacturonic acid gamma-lactone, i-erythritol, D-xylose, N-acetyl-D-glucosamine, D-cellobiose, and alpha-D-lactose), phenolic compounds (2-hydroxybenzoic acid and 4-hydroxybenzoic acid) and polymers (Tween 40, Tween 80, alpha-cyclodextrin, and glycogen). For each series, the corrected absorbance values of the substrates were summarized and expressed by the mean corresponding to each of the guilds.

### 2.4. Soil physical and chemical factors

The determination of chemical and physical factors was performed for each of the 36 soil samples. Soil pH was mensured using a soil suspension in 0.01 M CaCl_2_ (1:5, w/v). Soil moisture was determined by the gravimetric method, as described by the Brazilian Farm Research Corporation (Silva, 2009). The total carbon (C) and nitrogen (N) contents were determined by dry combustion using a CHNS/O elemental analyser (PerkinElmer, Waltham, MA, United States) from the soil samples. The determination was performed using 5–7 mg of dry soil sieved through a 0.15 mm mesh. The soil NH_4_^+^-N and NO_3_^−^-N were extracted with 2 M potassium chloride (KCl) and quantified using spectrophotometry as described by Norman et al. (1985) and (Krorn, 1980), respectively.

### 2.5. Statistical analysis

The absorbance data from 168 h were used for analysis of AWCD and metabolic richness using the “pacman” package (Rinker and Kurkiewicz, 2017) of the R software for the Mann–Whitney statistical test. The Tukey test was used to determine differences between the soil samples from the three floodplains with different water types in the Amazon for AWCD and metabolic richness. Heatmaps were generated based on the average AWCD values at 168 h using the R software package “pheatmap” (Kolde, 2019). The physical and chemical factors of the soil were subjected to Tukey’s test to evaluate the differences between the land uses in each floodplain and seasonal period. Redundancy analysis (RDA) was used to evaluate the relationship between microbial function and environmental factors and was performed using the program Canoco 4.5 (Biometrics, Wageningen). In addition, to quantify the contributions of “seasonality” (flood pulses), “land use” and “water type” to the consumption of functional guilds, variance partitioning analysis (VPA) was performed with the “vegan” package of R software (Oksanen et al., 2016). Statistical analyses were performed using “R Studio 3.5.1” software (R Core Team, 2018).

## 3. Results

### 3.1. Analysis of the physiological profile at the community level

**Figure 2:**
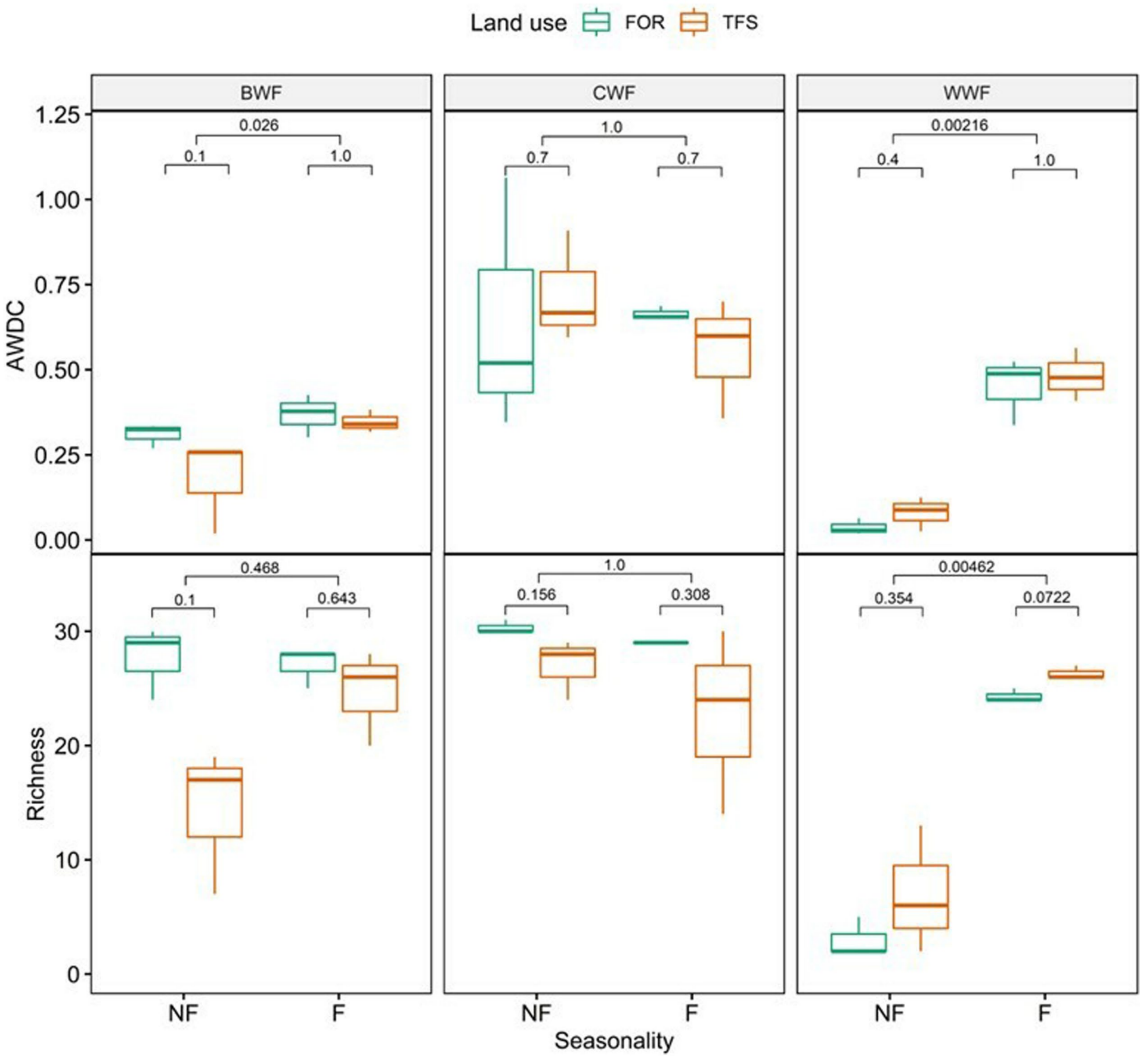
Average well color development (AWDC) and richness of substrate use by the microbial community in floodplain Amazonian soils flooded with black water (BWF), clear water (CWF), and white water (WWF) under different land uses (FOR, forest and TFS, traditional farming system) and sampled in two seasonal periods (NF, not flooded and F, flooded) based on the use of carbon sources 168 h after incubation in BIOLOG™ EcoPlate. The bars accompanied by values indicate the value of *p* obtained by Mann–Whitney test.

AWCD and richness of substrate use by the microbial communities in the soils from the forest sites and the traditional farm systems of the black, clear, and white water Amazonian floodplains 168 h after incubation in the BIOLOG™ EcoPlate are shown in Figure 2. For both variables, there was no significant difference between the values at the forest sites and the traditional farm systems for the three floodplains studied. AWCD showed differences in relation to seasonal periods in the floodplain soil of the black (*p* < 0.05) and white (*p* < 0.01) rivers (Figure 2). In both of these floodplains, an increase in AWCD was occurred during the flooded period.

**Figure 3:**
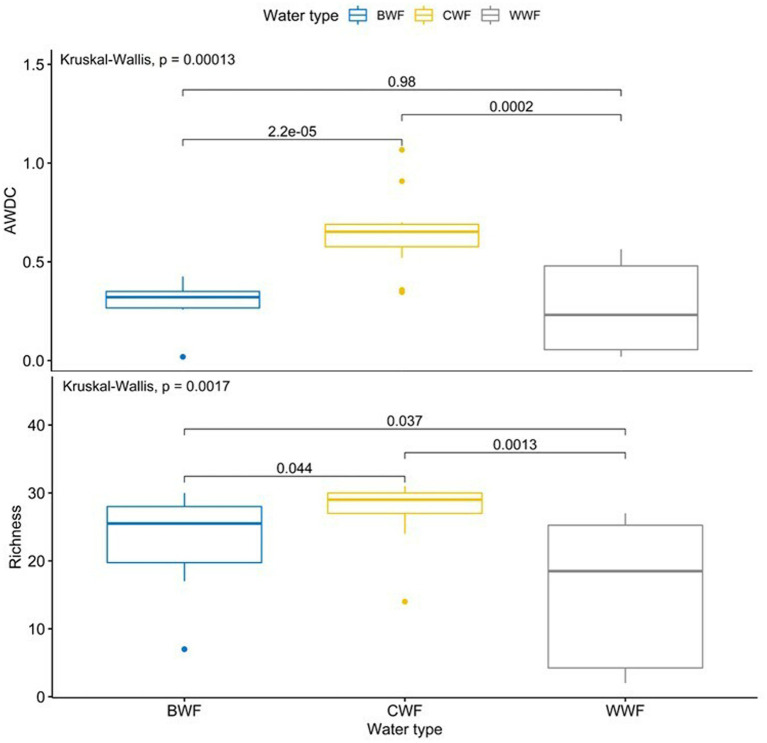
AWDC and richness of substrate utilization based on the use of carbon substrates in 168 h by the microbial community in soils from Amazonian black, clear and white water floodplains. Water types are abbreviated as follows: BWF, black water floodplain, CWF, clear water floodplain, WWF, white water floodplain. Bars accompanied by values indicate the value of *p* obtained by the Tukey test.

To compare the metabolic diversity between the different conditions evaluated, the richness of substrate use was used and showed that in the white water floodplain, seasonality had the greatest influence on the metabolic diversity of the soil microbial community (*p* < 0.01) (Figure 3). The forest site (3.00 ± 1.73) and the traditional farm system (7.00 ± 5.56) in white water floodplain showed less substrate use in the non-flooded period than in the flooded period. Although the data on the richness of substrate use by the black water floodplain microbiota showed no significant differences, in comparison to the traditional farm system, the forest site showed greater substrate use richness in both periods. The richness of substrate use by microbial communities in forest soils of the clear water floodplain decreased from 30.33 (±0.57) to 29.00 (±0.00) with flooding. In the traditional farm system, the decrease was 27.00 (±2.64) to 22.67 (±8.08). Thus, the soil at the clear water floodplain forest site showed the highest richness of substrate use during the study.

The dataset on the soils of the three studied Amazonian floodplains was used to compare the differences in AWCD and richness of substrate use under the influence of the different water types. As shown in Figure 3, the AWCD was significantly higher in the soil of the clear water floodplain (0.64 ± 0.20) than in the black water floodplain (0.30 ± 0.10) and white water floodplain (0.26 ± 0.22). There was no significant difference between soils of the black and white water floodplains in relation to the AWCD. Regarding the richness of substrate use, the soils of the floodplains flooded with three water types showed a contrasting behaviour (*p* < 0.05): clear water floodplain > black water floodplain > white water floodplain (Figure 3).

**Figure 4:**
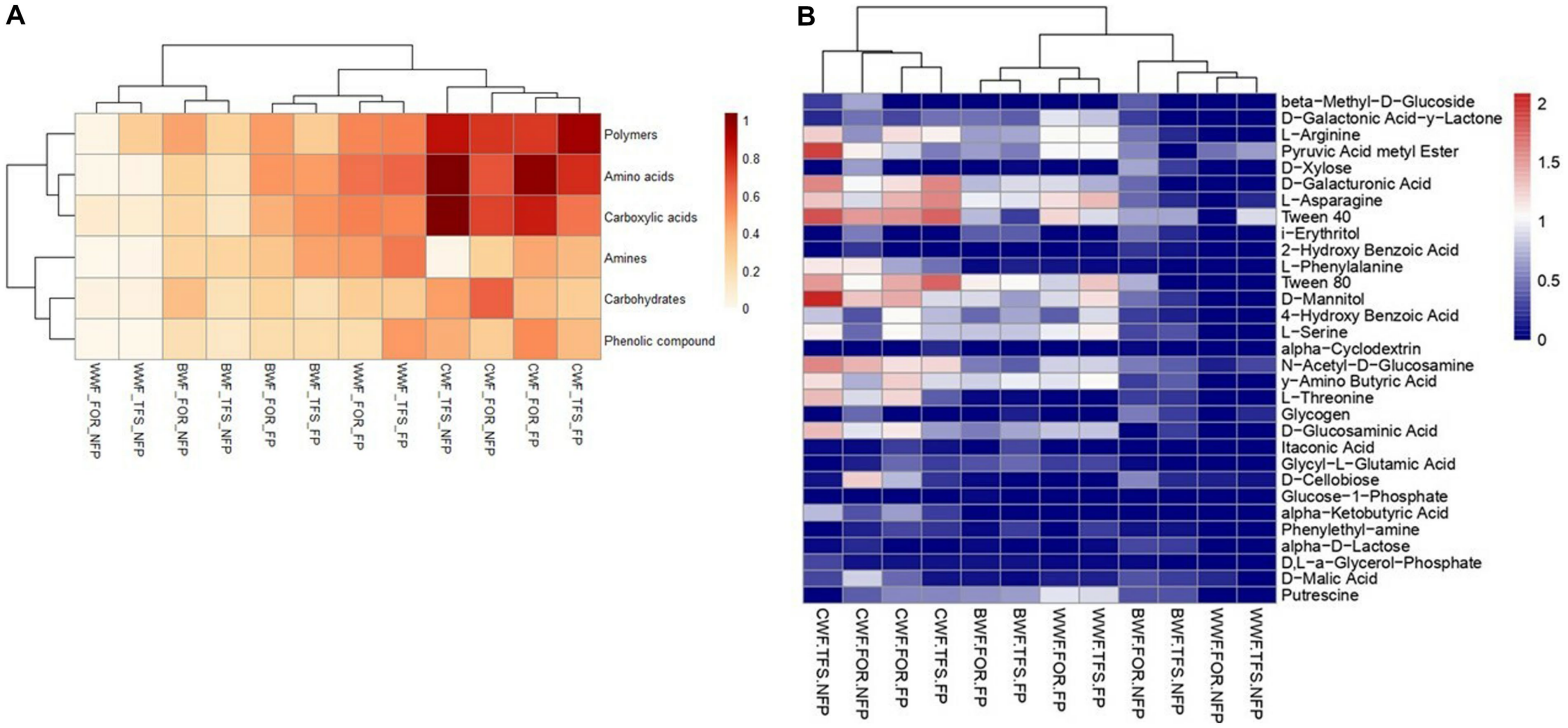
Heatmap showing carbon utilization response for the different substrate guilds—amino acids, amines, carboxylic acids, carbohydrates, phenolic compounds, and polymers and with dendrogram of the utilization of the 31 carbon sources by the soil microbial community in different floodplain Amazonian soils. Water types are abbreviated as follows: BWF, black water floodplain, CWF, clear water floodplain, WWF, white water floodplain. The different land uses (FOR, forest and TFS, traditional farming system) and sampled in two seasonal periods (NF, non-flooded and F, flooded) based on the use of carbon sources 168 h after incubation in BIOLOG™ EcoPlate.

The comparisons of the substrate guilds and the use of carbon sources by soil microbial communities at the forest sites and traditional farm systems in the floodplains of the black, clear, and white waters during the non-flooded and flooded periods are shown in Figure 4A. Considering the seasonal effect, the data obtained for the soils of the clear water floodplain showed different behaviour trends among the sampling sites. At the forest site, flooding resulted in increased use of the polymer guild for amino acids. Conversely, in the traditional farm system, the opposite trend was observed, with flooding resulting in a change from amino acids/carboxylic acids to polymers. Comparing the distribution of the substrate guild use in the soils of the floodplains of the three water types, during the non-flooded period, the least used guild was amines, and the most widely used guilds were carboxylic acids, polymers, and amino acids.

The differences between substrate use are shown in Figure 4B. Soil samples collected in the white water floodplain during the non-flooded period were grouped given the overall low substrate use. The substrates that were most metabolized at each of the sampling sites had the following arrangement in relation to AWCD: Tween 40, 0.68 and D-xylose, 0.67 (FOR-NF), Tween 40, 0.71 (TFS-NF), Tween 80, 1.10, and L-asparagine, 0.98 (FOR-F), and gamma-aminobutyric acid, 0.98 and L-asparagine, 0.94 (TFS-F) were used as mentioned in forest and traditional farming system during the flooded and non-flooded periods in the black water floodplain. In turn, in clear water floodplain for both land use systems and seasonal periods, Tween 40, 1.51 (FOR-NF), D-mannitol, 2.08 (TFS-NF), Tween 40, 1.55 (FOR-F), Tween 40, 1.77, and Tween 80, 1.76 (TFS-F) were used. Finally, during the non-flooded period in the white water floodplain, pyruvic acid methyl ester, 0.50 (FOR-NF), and Tween 40, 0.91 (TFS-NF) were used, and in FOR-F (Tween 40, 1.25 and L-asparagine, 1.19), and TFS-F (L-asparagine, 1.33 and Tween 80, 1.28) Tween and L-asparagine were used.

A low metabolism rate or no substrate use was observed for almost all samples (Figure 4B), with the following arrangement: itaconic acid, 0.007 (FOR-NF), D-galacturonic acid, glucose-1-phosphate, and alpha-ketobutyric acid, 0.00 (TFS-NF), D-xylose and 2-hydroxybenzoic acid, 0.00 (FOR-NF), 2-hydroxybenzoic acid and alpha-ketobutyric acid, 0.00 (TFS-F) in black water floodplain. In turn, in clear water floodplain, glucose-1-phosphate, 0.017 (FOR-NF), D-xylose, 0.00 (TFS-NF), and 2-hydroxybenzoic acid, 0.00 (FOR-F and TFS-F). In white water floodplain, methyl ester of pyruvic acid, D-malic acid, D-cellobiose and N-acetyl-D-glucosamine showed metabolism < 0.10 (FOR-NF), methyl ester of acid pyruvic acid, L-asparagine, Tween 40, N-acetyl-D-glucosamine, glycogen and D-cellobiose showed metabolism < 0.10) (TFS-NF), D-xylose, 2-hydroxybenzoic acid, alpha-ketobutyric acid and phenylethylamine, 0.00 (FOR-F), and D-xylose and alpha-ketobutyric acid, 0.00 (TFS-F).

### 3.2. Contribution of soil physical and chemical factors to the variation in the functional metabolic characteristics of the bacterial community

**Figure 5:**
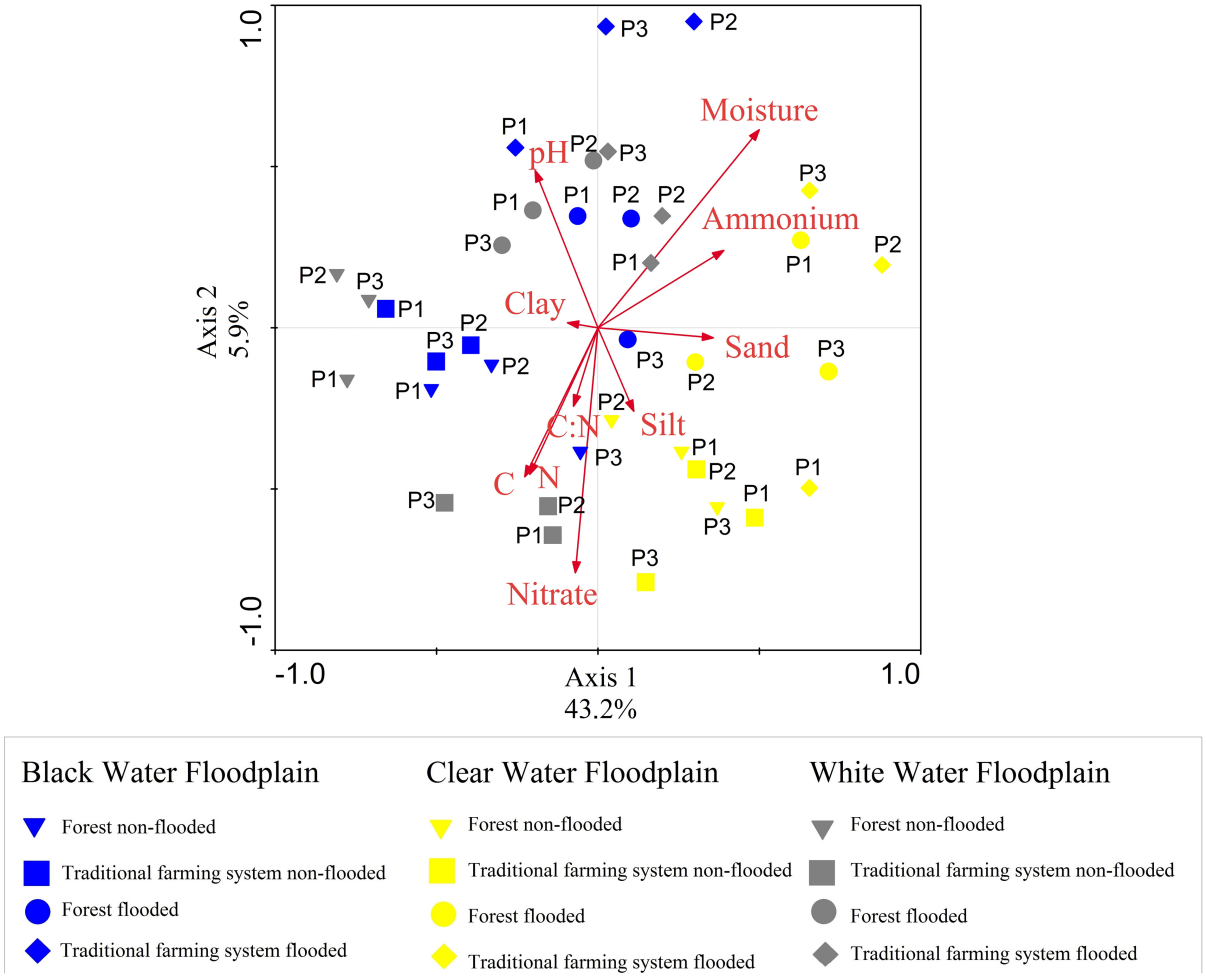
Restricted ordination diagram for the first two axes of redundancy analysis (RDA) based on soil physicochemical factors and their relationship with the utilization of carbon sources at 168 h by the microbial community at three sampling points (P1, P2, and P3) of forest sites and traditional farm systems of Amazonian floodplain river black water (in blue), clear water (in yellow) and white water (in grey) at two seasonal periods (non-flooded and flooded). The four geometric shapes represent the non-flooded forest site (inverted triangle), traditional non-flooded farming system (square), flooded forest site (circle) and traditional flooded farming system (rhombus).

The soil physical and chemical factors are shown in Table 1. RDA (Figure 5) was used to illustrate the relationship between the soil physical and chemical factors and the bacterial metabolic diversity. The explanatory variables, including relative humidity, total nitrogen, total carbon, ammonium, nitrate, pH, silt, clay, sand, and the C:N ratio, represented 49.10% in the first two axes of analysis. Moisture and nitrate were the main factors influencing metabolic activity in Amazonian floodplain soils.

**Table 1:** Physical and chemical factors in the soils from forest and traditional farm systems during non-flooded and flooded periods in Amazonian floodplains under different water types.

Physicochemical factors	Black-water river floodplain				Clear-water river floodplain				White-water river floodplain			
	NF		F		NF		F		NF		F	
	FOR	TFS	FOR	TFS	FOR	TFS	FOR	TFS	FOR	TFS	FOR	TFS
Moisture (%)	4.88^a^*a ± 2.18^b^	1.98*a ± 0.34	42.97*a ± 12.04	30.78*a ± 5.16	8.05*a ± 5.24	6.09*a ± 1.16	33.74*a ± 2.78	31.0*a ± 3.75	4.60*a ± 0.44	2.85*a ± 0.07	37.92*a ± 0.20	32.99*a ± 0.70
pH (CaCl_2_)	3.77*a ± 0.14	4.05*a ± 0.08	3.79*a ± 0.22	4.42*b ± 0.35	3.65*a ± 0.01	3.97*a ± 0.04	3.75*a ± 0.01	4.08*a ± 0.09	4.15*a ± 0.05	4.08*a ± 0.04	4.58*a ± 0.25	4.43*a ± 0.06
Total carbon (%)	4.45*a ± 1.80	4.55*a ± 0.59	2.12*a ± 0.64	0.96*a ± 0.64	2.05*a ± 1.04	1.37*a ± 0.14	1.49*a ± 0.69	1.14*a ± 0.85	1.35*a ± 0.10	1.12*a ± 0.21	0.75*a ± 0.25	0.86*a ± 0.35
Total nitrogen (%)	0.52*a ± 0.12	0.43*a ± 0.06	0.16*a ± 0.04	0.08*a ± 0.05	0.25*a ± 0.06	0.16*a ± 0.10	0.12*a ± 0.05	0.10*a ± 0.05	0.23*a ± 0.02	0.20*b ± 0.03	0.09*a ± 0.02	0.09*a ± 0.02
Ammonium (mg kg^−1^)	24.88*a ± 10.28	10.86*a ± 5.81	77.69*a ± 40.75	20.00*b ± 7.92	6.08*a ± 2.85	13.00*a ± 9.21	31.44*a ± 17.96	24.35*a ± 5.28	4.41*a ± 1.91	8.44*a ± 2.79	23.18*a ± 1.67	12.85*a ± 0.73
Nitrate (mg kg^−1^)	2.73*a ± 4.41	1.90*a ± 0.86	0.09*a ± 0.16	0.00*a ± 0.00	9.33*a ± 2.03	4.20*b ± 1.52	0.25*a ± 0.20	0.07*a ± 0.06	0.66*a ± 0.77	7.10*b ± 2.38	0.00*a ± 0.00	0.00*a ± 0.00
Sand (%)	63.43 ± 7.65	77.64 ± 6.67	63.43 ± 7.65	77.64 ± 6.67	30.14 ± 2.42	53.39 ± 14.92	30.14 ± 2.42	53.39 ± 14.92	2.84b ± 0.24	8.11a ± 2.81	2.84b ± 0.24	2.84b ± 0.24
Clay (%)	29.35 ± 5.08	18.07 ± 5.02	29.35 ± 5.08	18.07 ± 5.02	26.03 ± 3.34	17.62 ± 8.34	26.03 ± 3.34	17.62 ± 8.34	22.33 ± 1.20	21.8 ± 2.10	22.33 ± 1.20	22.33 ± 1.20
Silt (%)	7.22 ± 2.60	4.3 ± 1.66	7.22 ± 2.60	4.3 ± 1.66	43.83 ± 1.25	28.99 ± 11.5	43.83 ± 1.25	28.99 ± 11.5	74.83a ± 1.40	70.09b ± 0.78	74.83a ± 1.40	74.83a ± 1.40

FOR, forest site; TFS, traditional farm system. NF, non-flooded period; F, flooded period. Values with the same letters were not significantly different (*p* < 0.05) based on upon a Tukey’s HSD test. Tukey’s test was performed contrasting FOR vs. TFS within each floodplain and seasonal period for soil. Significance levels: *p* > 0.05; *p < 0.05.

a Average for each of three replicates of soil.

b Standard deviation of the average for each of three replicates of soil.

### 3.3. Contribution of seasonality, land use type, and water type to the variation in the metabolic characteristics of the microbial community

**Figure 6:**
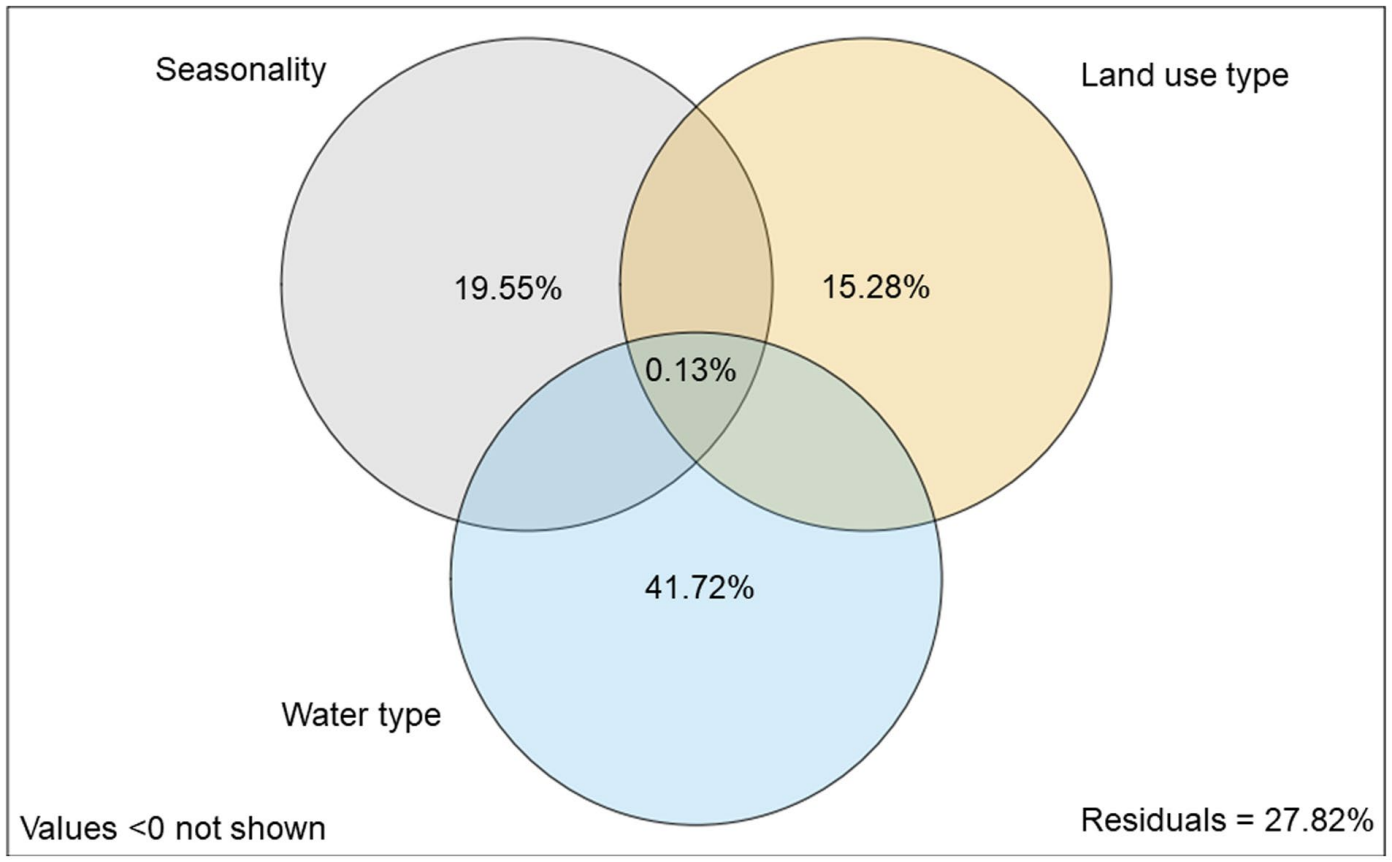
Variance partitioning analysis (VPA) of the contribution of seasonality, land use and water type to the variation in the consumption of substrate guilds (amino acids, amines, carboxylic acids, carbohydrates, phenolic compounds, and polymers) by the microbial community in floodplain soils from different Amazonian waters.

The VPA (Figure 6) was used to clarify the influence of seasonality (non-flooded and flooded period), land use (forest and traditional farm system), and water type (black, clear, and white) on the metabolic function (functional guilds) of the microbial community. The analysis revealed that the proportion of the metabolic activity of the microbial communities explained solely by the water type (41.72%) was greater that explained by the variations in seasonality (19.55%) and land use type (15.28%).

## 4. Discussion

BIOLOG™ EcoPlates have been increasingly used in approaches for evaluating the metabolic profiles of microbial communities in wetlands (Lv et al., 2017; Furtak et al., 2019; Zhang et al., 2019; Ramírez-Vargas et al., 2020; Zhen et al., 2020; Wang et al., 2021). This study revealed the distribution of the metabolic functional diversity of microbial communities in different Amazonian floodplains for the first time. We emphasize that the BIOLOG™ EcoPlate methodology has a closed configuration, and the response achieved is within an expected spectrum, i.e., within the sources studied by the methodology. Our data are of an exploratory nature and highlight the need to learn more about the physiology and ecology of Amazonian soil microbiota.

### 4.1. Influence of land use type on metabolic diversity patterns

The microbiota inhabiting soil from forest site in the black water floodplain revealed overall ability to use a greater range of carbon sources (richness), and higher AWCD in comparison those from the traditional farm system site. In turn, the soils from traditional farm system of the clear water floodplain showed a higher overall utilization capacity in comparison to the forest site in the non-flooded period; however, its metabolic richness was lower. In the flooded period, these soils in the forest site responded with the highest metabolic activity and richness of substrate use. Based on the microbial community of the white water floodplain soil, the traditional farm system had higher microbial activity and metabolic richness than the forest site in both periods. Ecological theory suggests that greater plant diversity should lead to greater functional diversity of a microbial community; thus, it is surprising that the influence of plant diversity on the function of the microbial community has not been widely observed (Button et al., 2016; Wu et al., 2021) in all floodplains. In addition, Lagerlöf et al. (2014) emphasize that the technique may favors *r*-strategist microorganisms, i.e., those that grow rapidly in substrates, rather than *k*-strategist microorganisms, i.e., oligotrophic species that are at a disadvantage.

Regarding the difference in the specific use of organic sources, some inferences can be made in relation to land use. Amines (phenylethylamines) were not used by the soil microbiota of the white water floodplain at the forest site. Oest et al. (2018), when investigating the patterns of carbon substrate use in the microbial sediment communities of the Saint Clair River and Saint Clair Lake on the border between the United States and Canada, observed that this amine had been used at a significantly higher level in the sediments of the river and were not used, or were poorly metabolized, by microbial communities in the lake sediments.

During the non-flooded period, D-xylose, which is the second most abundant sugar of lignocellulose (Nakamura et al., 2008), was not used by the soil microbiota in the traditional farm system site from clear water floodplain, whereas at the forest site of the same floodplain, D-xylose was moderately consumed with an AWCD of 0.64.

### 4.2. Influence of flood pulses on metabolic diversity patterns

Flooding can be considered a major disturbance in floodplain ecosystems and can affect carbon dynamics and microbial dynamics (Wilson et al., 2011). Most of these changes may result from the release of soluble organic compounds in the soil, creating favorable conditions for the growth of microorganisms (Furtak et al., 2020).

The analysis of the substrate use data for the 31 carbon sources revealed that flooding resulted in a more diverse and intense range of substrate use by the native microbial population. However, some sources showed behaviors that are worth highlighting, such as the soils of the black and clear water floodplains not using the phenolic compound 2-hydroxybenzoic acid during the flood period. In a study conducted by Furtak et al. (2020) with a flood simulation, this substrate was one of the least degraded by the soil microbiota. Rutgers et al. (2016) indicate that this substrate is difficult to degrade. In contrast, the evaluated phenolic compound (4-hydroxybenzoic acid), which is distinguished by the position of the hydroxyl groups in the phenol ring, was well- metabolized during the flooded period. Among the carboxylic acids that showed changes between the periods, d-galacturonic acid was well assimilated by the microbiota present in the soil in the black, clear, and white-water river floodplains during the flood period. Furtak et al. (2020) showed that d-galacturonic acid is one of the most intensely decomposed substrates. According to Salomo et al. (2009), d-galacturonic acid generally appears during the enzymatic hydrolysis of pectin.

Although the use rate of the six carbon guilds by the soil microbial communities varied with water type and seasonal period, the preferred groups were amino acids, polymers, and carboxylic acids. Among the six amino acids evaluated, the best metabolized substrate during flooding due to the flood pulse was L-arginine, L-asparagine, and L-serine. L-asparagine and L-serine belong to the group of polar amino acids and have similar chemical structures (Salomo et al., 2009). Soil organic matter accumulation and higher soil N content were positively correlated with the addition of free, adsorbed, and hydrolyzable amino acids in high-altitude soil (Cao et al., 2021). Thus, the entry of residues and the likely increase in litter level resulting from flood pulses may result in these substrates being more commonly used. The production and use of amino acids by plant or microbial cells, as well as ecosystem processes, are all significantly related to the composition of soil amino acids (Cao et al., 2021).

The use of polymer substrate groups was maintained at a higher level during the non-flooded period. The creation of a more favorable environment for certain soil microorganisms to use specific substrates was also observed by Feigl et al. (2017) when monitoring the changes in a microbial community in degraded acid soils, which were evaluated in a microcosm experiment using red mud bauxite as a possible stimulating agent for microbial activity.

Christian and Lind (2006), when evaluating seasonal patterns in the sediment–water interface in a eutrophic reservoir in the United States, reported seasonal differences in the pattern of substrate use. During the spring, a period in which there was an increase in temperature and a decrease in dissolved oxygen (DO), the preference was for carbohydrates. With the beginning of summer, a greater increase in temperatures and the depletion of DO, the preference of substrates changed to amino acids, carboxylic acids, and amines. Conversely, during the winter, carbohydrates and polymers were the most metabolized. Sediment from a tropical freshwater stream evaluated under aerobic and anaerobic conditions showed higher metabolic diversity under anaerobic conditions (Costa et al., 2015).

In soils of the black and clear water floodplains, carbohydrate D-xylose was preferentially used at the forest sites during the non-flooded period. Under the influence of flooding, there was a marked decrease in this substrate, or it was not used. A decrease in use was also observed for other carbohydrates, such as D-cellobiose, alpha-D-lactose, and beta-methyl-d-glucoside.

The Tween 40 polymer was the most metabolized substrate in general. Except for low use by the soil microbiota in the traditional farm system of the black water river and the clear water river forest site during the non-flooded period, the AWCD was above 0.65 at all sampling points. When evaluating the structural and metabolic diversity of microbial communities of the rhizosphere of *Phragmites karka* in a tropical coastal lagoon in India, Behera et al. (2018) found that Tween 40 and 80 polymers were among the nine carbon sources used in all sediment samples. The authors reported the highest consumption of carbon substrates: D-galacturonic acid (carboxylic acids), 4-hydroxybenzoic acid (phenolic compound), D-mannitol (carbohydrate), L-asparagine (amino acid), putrescine (amines), and Tween 40 (polymer). Our results show a similar pattern of behaviour for the substrates Tween 40 and 80 (polymers), L-asparagine (amino acid), and D-mannitol (carbohydrate). Thus, polymers and amino acids that are typically present in root exudates (Behera et al., 2018) seem to provide the best growth substrates for Amazonian floodplain soil microbial communities.

### 4.3. Influence of water colour on metabolic diversity patterns

The responses of the microbial communities in the floodplain soils of the black and white water floodplains showed the same pattern in relation to a lower use of substrates (phenolic compounds). The data suggest that soils under the influence of clear water have less ability to use amines during the non-flooded period and carbohydrates during the flooded period. In a recent study conducted in Chinese tropical mangroves, the authors traced the use profile of carbon sources analysed by the BIOLOG™ EcoPlate. The utilization rate of polymers, amino acids, and carboxylic acids varied according to the sampling region but was higher than that used for the other three carbon sources (Wang et al., 2021).

Clearly, understanding how microbial communities respond to climate change vectors under field conditions is important for accurately predicting how ecosystems can respond to future climate scenarios. Castro et al. (2010) evaluated the responses of a soil microbial community to multiple experimental factors of climate change, and precipitation had the greatest impact on the composition of the microbial community. The authors emphasize that soil moisture can affect the physiological state of microorganisms, soil physicochemical properties, and plant productivity.

The environmental factor ‘soil moisture’ of the floodplains of the black and white water floodplains increased the metabolization of carbon sources and changes in the use of guilds, whereas in the floodplain soils of the clear water floodplain, the flood pulse was a predictor of changes in guilds used. During the flooded period, the amino acid guild, which is considered a nitrogen-rich carbon source for plants in natural ecosystems and farm systems, was primarily used (Chapin et al., 1993; Kielland et al., 2006). The microbiota, in addition to using the carbon present in such substrates, incorporated the NH_4_^+^ side chain, which was later transformed into organic molecules, such as amino acids and proteins (Oest et al., 2018). Liu et al. (2017), when evaluating rates of absorption and contributions of organic and inorganic forms of N by labelling dominant tree species in temperate and tropical forests with ^15^N, revealed a strong preference for NH_4_^+^ rather than glycine and absorption of NO_3_^−^. The data for the nitrogen fractions showed an increase in the NH_4_^+^ concentrations due to the flood pulse, according to Cusack et al. (2011) the increase in nitrogen (N), which particularly occurs in tropical forests rich in N, and this increase can probably change the composition and behaviour of microbial communities based on the complex interactions between microbial community composition, enzymatic capacity, and soil chemistry.

## 5. Conclusion

In conclusion, this study showed that the changes in the soil resulting from the flood pulses and the consequent increase in the soil moisture triggered an increase in metabolic activity in the soils of the black and white water floodplains. In contrast, in the soil from clear water floodplain, with higher overall metabolic ability, the shifts by flood pulses were followed by changing from a higher consumption of polymers and carboxylic acids to a higher consumption of carbohydrates in forest site and from a higher consumption of amino acids and carboxylic acids to a higher consumption of polymers in traditional farm system. Hence, our results support the hypothesis that flood pulses in Amazonian floodplains affect the ability of soil microbial communities to metabolize organic substrates. Regarding the second hypothesis, the results showed that there are differences in the microbial community-level physiological profiles of soils under the influence of different Amazonian water types. We demonstrated that the metabolic richness of the soil microbiota in the white water floodplain was distinct from that in the black and white water floodplains with the microbial communities exhibiting distinct physiological profiles based on a variety of carbon sources. We thus provide information that reinforces the importance of including soils under the influence of flood pulses, water types, and land use as environmental factors when recognizing functional diversity and ecosystem functioning in Amazonian floodplains.

## Data availability statement

The original contributions presented in the study are included in the article/supplementary material, further inquiries can be directed to the corresponding author.

## Author contributions

DB, ST, and AN conceived and designed the study. DB, MC, GC, and VN performed the research. DB and LV analyzed the data. DB, DK-A, EG, PM, ST, and AN wrote the paper. All authors contributed to the article and approved the submitted version.

## Funding

This study was financed by a grant from Fundação de Amparo à Pesquisa do Estado de São Paulo, Brasil (FAPESP) (2016/16687-3). DJB was supported by the Coordenação de Aperfeiçoamento de Pessoal de Nível Superior, Brasil (CAPES), Financial Code 001. MC was supported by the Conselho Nacional de Desenvolvimento Cientıifico e Tecnológico, CNPq, Brazil (Grant Number: 142146/2017-0) and CAPES (Finance Code 001). AAN was supported by FAPESP (2017/03575-5). AAN and TSM would like to thank CNPq for the Research Productivity Grants (309746/2021-3 and 314806/2021-0).

## Conflict of interest

The authors declare that the research was conducted in the absence of any commercial or financial relationships that could be construed as a potential conflict of interest.

## Publisher’s note

All claims expressed in this article are solely those of the authors and do not necessarily represent those of their affiliated organizations, or those of the publisher, the editors and the reviewers. Any product that may be evaluated in this article, or claim that may be made by its manufacturer, is not guaranteed or endorsed by the publisher.
